# Exploration of perceptions and decision-making processes related to childbirth in rural Sierra Leone

**DOI:** 10.1186/s12884-015-0500-9

**Published:** 2015-04-08

**Authors:** Laura Treacy, Mette Sagbakken

**Affiliations:** International Community Health, University of Oslo, P.O box 1130, Blindern, Oslo, 0317 Norway; Oslo and Akerhus University College, Faculty of Health Sciences, Oslo, Norway; National Center for Minority Health Research (NAKMI), Gullhaugveien 1-3, 0484 Oslo, Norway

**Keywords:** Childbirth, Rural health, Decision-making and perceptions

## Abstract

**Background:**

Maternal mortality ratio (MMR) remains high in Sierra Leone. Efforts have been made to reduce MMR by increasing the number of women delivering at a health facility through introduction of the Free Health Care Initiative in 2010. Despite this, utilisation remains lower than aimed for, with marked inequalities between rural and urban settings. This study explores the perceptions and decision-making processes of women and their communities during childbirth in rural Sierra Leone.

**Methods:**

A qualitative, cross-sectional study employing focus group discussions, in- depth interviews and informal interviews with pregnant women and community members in rural northern Sierra Leone. Data were analysed using systematic text condensation.

**Results:**

Data revealed that the decision-making processes are complex and multi-faceted. Decisions regarding the place of delivery and with whom assisting the birth are often made collectively. A normal delivery is seen as one that occurs within the village. Previous experiences, perceptions and expressions of bodily symptoms as well as the interpretation of different risks affect these decisions. The health seeking behaviours were found to be flexible and dynamic, and the final decisions about where to give birth could be governed by unexpected circumstances.

**Conclusions:**

Decision-making processes during childbirth in rural Sierra Leone are dynamic and intricate and need to be understood within the broader social context. Future initiatives to improve access and utilisation of safe health services for pregnant women within rural Sierra Leone need to be based on adequate knowledge of women’s preferences, cultural-specific traits, capabilities, perceptions of risk and the constraints in which they may live.

## Background

Over the last 25 years there have been global efforts to improve maternal health, from the “Safe Motherhood Initiative” launched in Nairobi in 1987 [1] to the fifth Millennium Development Goal “Improve Maternal Health” in 2000 [2]. Maternal mortality, however, remains an international public health problem with an estimated 292,982 maternal deaths occurring in 2013 [3]. Maternal deaths in developing countries are thought to account for 99% of all maternal deaths globally [4], with 56% of these occurring in Sub-Saharan Africa [4,5].

There seems to be an international consensus that increasing the proportion of women delivering with skilled attendance will improve maternal health by reducing mortality and morbidity during childbirth [2,6,7]. Within the literature, reference to skilled attendance is often tantamount to delivery in a health facility [8]. These health facilities should assist with healthy births, and include basic emergency obstetric care as well as having access to well-functioning referral level care [7,9-11].

### Maternal health care in Sierra Leone

The maternal mortality ratio (MMR) for Sierra Leone remains high at 622.6 per 100,000 live births [3], compared to the global MMR of 209.1 per 100,000 live births [3]. Oyerinde et al. recommended an increase in health facility delivery as among the measures to reduce maternal mortality [12]. This strategy was advocated by the government in 2009 [13,14].

Recent studies conducted in Sierra Leone identified prohibitive costs to be one of the main barriers for women accessing maternal health care and utilising facilities during childbirth [15-17]. In 2010, in response to these findings, the government of Sierra Leone introduced the Free Health Care Initiative (FHCI) for pregnant women, breastfeeding mothers and children under the age of 5 [18]. A local-level initiative has also been introduced in the form of ‘bylaws’ [15]. The bylaws are described as a way to stimulate ‘facility care’ , implying that women are required to attend antenatal care and give birth at a facility. The laws are put in place by local authorities (typically chiefs), and women, husbands and occasionally Traditional Birth Attendants (TBA) can be fined if they do not comply [15]. The role that these laws actually play in local communities remains unknown.

With the introduction of FHCI, health facility deliveries initially increased at a rapid rate, reaching 54% in 2010 [14] but with a minimal increase to 54.4% by 2013 [19]. These estimates remain lower than the Government’s target of 90% of births being in a health facility by 2015 [14]. Utilisation of health facilities differs between urban and rural areas: 68.1% and 49.7% respectively. These inequalities persist for delivery with a skilled attendant: 78.9% delivering with a skilled attendant in urban areas and 53.2% in rural areas [19].

Other countries that have implemented free or reduced costs have also found that this effort alone does not ensure access to maternal healthcare for all [20-22]. Studies have highlighted the importance of context-specific research when exploring reasons behind utilisation or non-utilisation of health facilities [20-22]. Researchers have increasingly considered the experiences, perceptions, preferences and perceived risks of the ‘service-user’ during pregnancy and delivery to gain a deeper understanding of both decision-making processes and health-seeking behaviour within specific contexts [23-27]. Studies conducted before the introduction of FHCI in Sierra Leone found that barriers to accessing care at health facilities included prohibitive and unreliable costs, geographic inaccessibility, distance, lack of transport, and lack of supplies and human resources [15-17].

A review of the literature shows that there is a need to explore who and what influences the decisions made by women and their communities within rural areas of Sierra Leone; the impact of perceived risks related to different options; where women prefer to deliver; and why utilisation of health facilities during childbirth remains low now that prohibitive costs have, in theory, been removed. The aim of this study is therefore to explore the perceptions and decision-making processes related to childbirth in rural Sierra Leone.

The study will discuss some of the findings in the light of Kleinman’s [28] concepts. Kleinman describes the health care system as consisting of three partly overlapping sectors: the popular sector (individual, family and community beliefs and activities), the professional sector (the organized and institutional parts of health care) and the folk sector (sacred or secular types of folk medicine). He uses socially constructed ‘explanatory models’ to describe how individuals and their network explain and understand illness and disease, and justify the treatment that they seek. These models help to explain questions regarding aetiology, pathophysiology, course and consequences of illness, and subsequent treatment choices [28].

## Methods

This was a qualitative, cross-sectional study using purposeful sampling to obtain maximum variation. Data were collected through focus group discussions (FGDs) and individual and group in-depth interviews (IDIs) with recently pregnant women and members of their communities. It was partly explorative (abductive) with interviews and conversations being informed by literature and recent initiatives, and partly by themes introduced by the participants in the field.

### Study area

This study was conducted in La-lenken section, Tonkolili district in the Northern Province of Sierra Leone. In 2013, 37.8% of the women in this district gave birth with a skilled provider and 35.2% in a health facility, both of which are lower than the national average [19]. La-lenken section has an NGO-supported governmental hospital: Masanga Hospital, which operates as both a hospital and a Primary Health Unit. It is staffed 24 hours, 7 days a week and provides comprehensive emergency obstetric and newborn care. The study was conducted from August to December 2013 in 2 rural villages within the La-lenken section. Both villages used Masanga Hospital for their main institutional health needs. The 2 villages differed to ensure variation with regards to: geographical site, population size and socio-demographic variables. The first village (A) was closer to the hospital, had a smaller population size (<200) and relied on subsistence farming. The second village (B) was further from the hospital, and had a larger population size (>500), which relied on both subsistence farming and gold mining.

### Participants

In total 61 participants were included in this study. At each village three FGDs were conducted at the beginning of the data collection period with one additional one at the end. This final FGD was conducted to increase the validity of the initial findings and to see if any new themes emerged. Ten individual IDIs were conducted with women who had been pregnant within the last year. Individual and group interviews were also held with what was identified as key informants including health workers, TBAs and a group of motorbike drivers. This last group were included because they often escorted pregnant women from the villages to Masanga Hospital. Seven participants were re-interviewed at least one additional time in order to clarify certain topics, or to cover emerging themes discussed in later interviews. FGD and IDI participants’ characteristics are displayed in Tables 1 and 2. Informal interviews with other key informants such as representatives from national and international non-governmental organisations working in women’s health; and representatives from the local radio station were conducted throughout the data collection period. This was done to gain further insight into the local discourse around women’s health.

**Table 1:** **Characteristics of participants in FGDs and IDIs**

**Focus group discussions**	**Gender**	**Village**	**No. of participants**
Women who had been pregnant within the last year	Female	A	7
		B	5
Older women involved in a delivery within the last year	Female	A	6
		B	7
Men with a close family member who had been pregnant within the last year	Male	A	4
		B	9
Women who had been pregnant within the last year (final FGD)	Female	A	4
**In-depth interviews**			
Women who had been pregnant within the last year	Female	A	5
		B	5
Traditional Birth Attendants	Female	A	1
		B	2
Village chief	Male	A	1
Health personnel at local hospital	Male	Masanga	2
	Female		2
Motorbike drivers	Male	Masanga	4
Repeat interviews			7
*Total number of participants*			61

**Table 2:** **Characteristics of women who had been pregnant within the last year in IDIs**

**Participant**	**Number of pregnancies**	**Location of childbirths**	**Marital status**
**1**	5	Village	Married (2nd wife in polygamous relationship)
**2**	5	Village & hospital	Widowed
**3**	5	Village & hospital	Married
**4**	5	Village & hospital	Married (1st wife in polygamous relationship)
**5**	9	Village, bush road & hospital	Married
**6**	2	Village	Married
**7**	2	Village & hospital	Married (2nd wife in polygamous relationship)
**8**	1	Village	Separated
**9**	4	Village	Separated
**10**	4	Village	Married

### Sampling strategy and data collection

Upon arrival in each village the research team (first author with research assistant) met with the village chief and other key persons, such as the youth leader or TBA. The research project was introduced and criteria for participants explained. A homogeneous sampling technique was used initially to identify participants for the FGDs [29]. Each FGD took between 30 and 90 minutes and was conducted in quiet, sheltered locations (village meeting place or the village church) on the peripheries of each village in order to ensure privacy and reduce interruptions.

Potential participants for the IDIs were approached either after the FGDs or whilst the research team walked around the villages, speaking to community members performing their daily activities. Participants were chosen for their ability to provide in-depth and personal knowledge on the topic. Purposeful sampling was conducted to ensure maximum variation, for example those who seemed outspoken or quieter; those who showed variation in appearance/dress; or those that came from different parts of the village. In addition, the TBAs or other well-known women in each village suggested specific potential participants who the research team may not have been able to identify. For example: women who had lost their child during or after childbirth; those who had a non-complicated versus complicated birth; or those whose husband was in a nearby location or not. This snowball sampling strategy was utilised to identify potentially ‘information rich’ participants as well as to ensure maximum variation. Each IDI was conducted in a location chosen by the participants themselves and lasted between 45 to 120 minutes.

The first author led both the FGDs and the IDIs using thematic guides with the research assistant translating between English and Temne (local language). The questions or wording were adapted depending upon the participant or group being interviewed, with additional prompts to assist with quiet or shy participants. The main themes initially discussed were: experience of pregnancy and labour; who was involved during the delivery process and their roles; differences between delivering in the village and the health facility; risks or problems during pregnancy and childbirth; knowledge of the free health care initiative; knowledge of the bylaws. The interview and discussion guides were adjusted during the data collection with themes that emerged being included and explored in subsequent interviews. During the interviews and discussions the research assistant translated questions and short answers verbatim, whilst longer descriptions or explanations were summarised. The key informants were interviewed either at their work places, in the village or near their homes.

### Data analysis

All FGDs and IDIs were audio-recorded and transcribed by the first author in the field. The research team read these transcriptions, along with summaries of each FGD/IDI, whilst listening to the audio-recordings. Mistakes or misunderstandings during the original interview translation were identified and corrected. In addition, parts summarised during the interviews were then expanded so that the participants’ original words and descriptions were transcribed verbatim. A number of audio-recordings were re-translated by an independent person at regular intervals during the fieldwork. This enabled both translations and cultural meanings behind the various discourses to be checked, providing confidence in the quality of the interpretation of data.

Preliminary analysis was conducted throughout the fieldwork period. A field diary, conceptual maps and daily reflections all assisted to identify emerging themes, and adapt the interview guides. Data concerning factors that affected the perceptions and decision-making processes during childbirth was used for systematic text condensation, according to the principles of Giorgi’s phenomenological analysis [30], modified by Malterud [31]. The analysis followed these steps (i) gaining an overall impression of the material by reading all of the data collected, (ii) identifying and sorting the material into codes, (iii) condensing the codes to themes, and (iv) analysing these themes so that descriptions and concepts are formed [31]. Each transcript was coded using the qualitative analysis software NVivo10. Themes were identified from the codes, with both codes and themes being continuously reflected upon, adapted, subgroups formed, changed and merged as appropriate. Finally each transcript, with all the identified codes and themes, were printed and re-read again. Codes and themes were analysed and described, highlighting the similarities and differences amongst the material, the participants and the different participant groups. Table 3 presents a summary of the themes and subthemes identified and analysed.

**Table 3:** **Themes and subthemes identified during the analysis process**

**Theme**	**Subthemes**
Home delivery as the norm	• Perception of the hospital
	○ Perceived role of the hospital
	○ Fear of the hospital
	• Previous experience and outcomes
Collective decisions	• The role of the pregnant woman
	○ 1st time mothers
	○ Multigravida mothers
	• The influence of female family members
	• The role of male family members
	• The influence of the TBA
Perceptions and expressions of bodily symptoms	• Symptoms motivating different types of treatment
	• Bearing up with the pain
	○ The value of being a strong woman
	○ Preventing a crowd
	• Perceptions of risk
	○ Immoral behaviour and complications
	○ Fear of witchcraft
Role of God	• Explanation of problems/complications
	• Fatalistic approach
	○ Marked by God
	• Justification of actions
Flexibility and dynamics in health seeking	• Use & accessibility of ‘Oporto’ medicine
	• Use & accessibility of traditional medicine
	• Free Health Care Initiative
	• Bylaws

### Ethical considerations

The Ministry of Health Services of Sierra Leone and Norwegian Social Science Data Services approved the study. Local approval was also obtained from the La-lenken section and village chiefs to conduct the study in their villages. Approval from the Regional Committees for Medical and Health Research Ethics in Norway was not needed. Informed written or verbal consent was obtained from all participants. Confidentiality was ensured by the removal of names and other identifying-information from transcriptions and analyses. Participants were informed that they could withdraw from participation at any time, for any reason, without negative consequences.

## Results

The decision-making processes during childbirth in rural Sierra Leone are multi-faceted, complex and constantly changing. This manuscript focuses on the socio-cultural elements of these processes as identified through analysis of the data. Five themes emerged from this analysis: Home delivery as the norm; Collective decisions; Perceptions and expressions of bodily symptoms; Role of God; Flexibility and dynamics in health seeking.

Additional findings relating to structural factors such as direct and indirect costs, perceived accessibility to health services, and the role of men and women in society all add further dimensions to what can be described as a dynamic health seeking process. These data will be presented elsewhere.

### Home delivery as the norm

The majority of participants in this study described a safe delivery as one that occurred at home in the village. The hospital was predominantly seen as a place to go only if a problem should occur. Many participants subsequently viewed attending the hospital as a preventative measure for potential problems as unnecessary, as highlighted in this quote:

*“(…) Because they are not used to that. It is normal, even to us, to wait until you are sick before you go to the hospital (laughing). So the same thing with those people, it is like they say ‘until I have a problem I will not go to the hospital’ , they will see it as a waste of time”.* (Community health officer)

Many women articulated concerns that attending the hospital with a complication automatically resulted in an operation. This could create fear of the hospital, as demonstrated in this quote:

*“Some of them are afraid to go to the hospital to deliver, they think that if they go to the hospital they will be operated on”.* (TBA)

Many of the women who spoke about the assumption of automatically being operated on had never attended the hospital themselves during childbirth. The information usually came from discussions with the older women or TBAs.

Previous childbirth experiences and outcomes influenced where women preferred to deliver and with whom assisting. Women expressed that if they had previously experienced uncomplicated home deliveries, they would plan for future births to also occur at home. Likewise, women who had a serious problem during previous deliveries were more likely to plan to go to the hospital with subsequent deliveries.

### Collective decisions

An individual rarely made decisions alone about where to deliver and with whom assisting. Instead, these decisions were made in conjunction with older women, especially the TBAs or female family members. Male family members were mostly responsible for providing money and transport rather than being involved directly in discussions. Decisions were usually socially negotiated, dependent upon community expectations as well as the relationships and positions of those involved.

The findings show that childbirth is a private and almost secret process in the villages. Those who had not experienced childbirth themselves were not allowed to be involved or assist during delivery. This included men and children, but also young women who have not yet had their own children. This implied that a woman (or girl) who was expecting her first child often had limited understanding about what would happen during the birthing process. This lack of knowledge about what to expect and what she should do, transcended to how she handled the initial labour pains and how much autonomy and authority she held concerning the decision-making process.

It was found that a primigravid woman might speak out about the onset of labour pain sooner than a woman who had given birth before. During an IDI one woman gave an account of how she was influenced by, and reliant on, the experienced women around her during the birthing process:

*“I really want the old woman to take me to the hospital to deliver, but when the other people told me to stay here, and wait for a time, that is why I stay. (…) I don’t have any understanding about delivering; they are the ones that have the experience. So anything that they tell me, I need to listen to them”.* (Recently pregnant woman IDI)

Multigravida women expressed having more control over their delivery, and were less likely to be influenced by others. According to most participants they were more likely to “bear up with the pain” and ask for help at a later stage, as illustrated in this quote:

*“(…) pregnant women who have delivered more than 3 children, they know the symptoms which come to them, that is why they bear up the pain before they expose it to somebody, (…) some of them when the pain starts, it is 2 days before they voice it out”.* (TBA)

The level of autonomy, authority and decision-making power that women felt they had varied greatly on an individual basis. Their experience as well as role within their own family, and within the community, often dictated how comfortable they were voicing their own opinions and having them listened to.

The TBAs themselves spoke about their “rights” to tell the pregnant women what to do during delivery, as explained in this statement below:

*“If I can't help the woman to deliver (…) I have the right to tell the pregnant woman to go with me to the hospital because, if any problems occur with that pregnant woman, the government will handle me”* (TBA)

‘Handle me’ implied that the TBA would be taken to the police station or disciplined for not referring the pregnant woman to the hospital. However, none of the TBAs spoke about this situation of ‘being handled’ actually occurring to them. At what point, or with what specific complications, the TBAs would take the pregnant woman to the hospital often remained vague in the discussions, despite much probing and clarifying questions. The health workers did not share the TBAs’ strong convictions that they would take a woman who was unable to deliver, or had complications, to the hospital in a timely manner. They described experiences of TBAs not recognizing problems or keeping the pregnant woman too long in the village, as revealed in this statement:

*“The TBAs keep the women there for 2 days, 3 days, without communicating to the people that there is a problem, assuming that the women would be able to deliver. (…) Most of the pregnant women who come into hospital (…) will tell you, ‘Nurse it's not me, they kept me there for 5 days, they said I will be able to deliver and nothing happens, only when they see that I am about to die they decided to come with me’”.* (Midwife)

The midwife continued to explain that often the family are reliant on the advice from the TBA regarding the progress of the labour. The family do not always know that there is a problem until it is too late to seek alternative help.

### Perceptions and expressions of bodily symptoms

The perceived cause and severity of a problem affected what type of treatment was sought and when. Some participants associated fits or convulsions during childbirth with the mother having a demon inside her caused by eating the wrong bush meat. Treatment for fits caused by a demon would be sought from a traditional healer within the village. However, not all women in the villages accepted that demons were the cause of convulsions. During one of the FGDs a woman described how she had also experienced convulsions during her childbirth, but understood this to be caused by her not attending the antenatal clinic, nor taking all the treatment advised during her pregnancy. Her family thus sought help at the hospital during her childbirth.

Some participants considered the hospital to be the best place to treat complications such as bleeding, as highlighted in this quote:

*“If I was here in the village giving birth and bleeding occurred, there is no way for the TBAs to help me solve the problem (…) But when I give birth in the hospital, if the bleeding occurs they will give me treatment to cure it”.* (Recently pregnant woman IDI)

However, a few women also considered bleeding as a condition that could be treated in the village, as expressed in the quote below:

*“If that problem (bleeding) occurs with you, if you give birth in the hospital, they will give you treatment to stop the problem. But if you give birth in the village, they will give you native herbs which will also solve that problem”.* (Recently pregnant woman FGD)

Many participants spoke about women ‘bearing up with the pain’ by keeping the onset of labour quiet or secret. Some referred to women who attended the hospital during childbirth as simply those who could not bear up with the pain, rather than having a problem, thereby associating ‘bearing up with the pain’ with being a strong woman. Others spoke about the shame caused by interpreting the pains incorrectly, as described below:

*“If you feel the pain and after, you explain yourself to the people, later (…) if it is not yet time to deliver, it is very shameful”.* (Recently pregnant woman FGD)

Many participants discussed ‘bearing up with the pain’ so as to prevent a crowd being drawn, relating this to the tendency that people would gossip about what they had seen during the delivery. Another reason for wanting to keep the number of people who knew that a woman was in labour to the minimum was associated with the fear that those with bad intentions might hear about her vulnerable position. Many participants spoke about the fear of someone halting the progression of labour, killing the baby or even the mother. This was usually related to ‘witches power’ as described in the statement below:

*“If you have been quarrelling with someone before, some of them have hated mind for you, as soon as they heard that “oh this woman she is in labour”, they go somewhere else and do witches power. That is why they don't want to circulate the information”.* (Recently pregnant woman IDI)

Due to this perceived risk many women would not share the onset of labour with others until the labour was well established. This also meant that they did not want to highlight their condition by attending the hospital. Bad intentions, predominantly from women, could be due to previous arguments, or because of jealousy, for example between women in polygamous marriages.

Yet another reason women kept the onset of their labour secret related to the fear of being accused of immoral behaviour if the labour was considered to be taking too long. Many participants in the villages spoke about infidelity as a potential cause for a delayed or obstructed birth as another man’s sperm had “contaminated” the pregnancy. In such cases the woman in labour needed to ‘speak out’ or confess to the adultery in order for the birth to proceed. This perception of risk relating to such immorality (rather than e.g. risk of an obstructed birth due to a small pelvis) was captured in this statement:

*“Yes, if the labour is long and she is delayed, they will say ‘she has something to say. She has been cheating on the husband’ (…). They will keep the woman and say ‘unless she says something, nobody talk to her or touch her’”.* (Midwife)

This midwife explained how the community might choose to seek assistance from a traditional sorcerer to ascertain if the obstruction was caused by infidelity, and if that was the case ensure that the woman confessed in order to give birth safely. This pressure to confess could delay communities in bringing a woman to the hospital to seek potentially life-saving medical intervention.

### Role of God

Many participants referred to the role of God as a way to explain problems during pregnancy or childbirth. Some participants elucidated that if God has marked you to die, it did not matter where you gave birth or with whom helping, you could not prevent death. This perception is highlighted in this quote from an IDI:

*“If God has marked you to die during that pregnancy, whatever situation you are in, or whatever you choose to do, if God has marked you to die you don’t have any other way”.* (Recently pregnant woman IDI)

This reflected that many individuals in the rural villages viewed potential complications during childbirth as being unavoidable. Therefore, choosing to access one health sector over another may not be considered as a critical decision, since God was present in all health sectors and ultimately decided the fate of the woman and her newborn child. Participants also described how God could mark you to deliver in a certain location, as explained by one male participant during a FGD:

*“We prefer our wives to give birth in the hospital, but if God marks them to give birth safely here (in the village), they will”.* (Man whose wife had been recently pregnant FGD)

God was therefore used as a way to justify an action or decision taken, which might have contradicted the official advice given by the Government to give birth at a health facility.

### Flexibility and dynamics in health-seeking behaviour

When discussing types of treatment for problems during pregnancy or childbirth, many women interchanged fairly easily between the traditional native herbs provided in the village, and the ‘oporto’ medicine typically provided in the hospital. Often equal value was placed on both types of treatment and the decision regarding which treatment to use depended on accessibility to that treatment. A woman in one of the FGDs explained in the quote below how she sought help in the hospital because the person who usually provided the native herbs was not in the village:

*“I went to the hospital because the one who knows the native herbs to stop that bleeding while I was pregnant, she was not around, and that is why I went to the hospital”.* (Recently pregnant woman FGD)

Some participants described circumstances where they would try one form of treatment in the village initially, and if it did not help they would access the hospital. This situation was revealed in this quote:

*“As for us we don’t have oporto treatment (in the village) (…) we give the native herbs to the pregnant woman for her to give birth safely, and if that pregnant woman does not give birth, we take her to the hospital”.* (Man whose wife had recently been pregnant FGD)

In some instances, oporto medicine could be obtained in the villages from tradesmen or peddlers. Two women shared their experiences of having an old man assess them during labour, offer advice, and provide them with tablets or injections. A man in the village being directly involved during labour is a unique finding within this setting since childbirth is predominantly seen as ‘women’s business’ in rural Sierra Leone. A benefit of obtaining treatment from these tradesmen is that they would come to the woman in labour, rather than her having to get on a motorbike and travel elsewhere for care. Also the cost of the treatment could be negotiated or credited.

Nearly all participants were aware of the Free Health Care Initiative (FHCI) at the hospital, but lack of trust as to whether services would actually be free or not was often raised. As captured in this statement:

*“The nurse who has been working in Masanga has gone, this one is a new nurse, we don’t know about her. The first nurse who stayed with the people at Masanga never took money from us. We don’t know for this present nurse”.* (Recently pregnant woman FGD)

Concerns about unreliable supply of medication; limited supply of equipment; lack of adequate hospital buildings and trained staff to cope with the increase in patient numbers since FHCI had come into place, were often discussed.

In contrast, many of the participants were not aware of the bylaws. Of the participants who had heard of the bylaws none had been personally affected by them. Therefore, the ‘bylaws’ or ‘threat of the bylaws’ did not seem to influence the decision-making processes during childbirth in this particular area of rural Sierra Leone.

## Discussion

This is the first qualitative study conducted after the introduction of the FHCI in rural Sierra Leone exploring perceptions and decision-making processes related to childbirth, pregnancy and use of maternal health services. The results demonstrate that the decision-making processes are dynamic and intricate, and need to be understood within the broader social context.

### Perceptions of normality

Community norms, social pressure, beliefs about childbirth and where to deliver can all have a strong impact upon individual health-seeking behaviour [32,33]. This study has shown that within the rural Sierra Leonean context a normal and safe childbirth is seen as one that occurs at home, a phenomenon supported by several other studies in Africa [16,24,34-36]. Preventative care seeking is not the norm. Treatment within the professional sector [28] is predominantly sought only if a problem has already arisen [20,34,37].

Previous experience and birth outcomes influence women in their decisions regarding location of childbirth and choice of assistance. Decisions are guided by prior contact with the different health sectors and the plethora of health providers. In their literature review Gabrysch and Campbell [8] found that previous use of a health facility meant that women were more likely to use the health facility with subsequent deliveries. Women in our study who had delivered at the health facility and planned to deliver there again were predominantly those who had experienced a complicated delivery requiring ‘hospital treatment’ to deliver safely, findings similar to a study in Timor-Leste [38]. It should be noted that planning to deliver at the hospital did not always result in that plan becoming a reality. Prohibitive factors such as inaccessible health facility and lack of money could strongly influence whether a woman attempted to access the hospital during delivery or not. These structural factors are not discussed in detail in this current paper but are known to affect health-seeking behaviours [39,40].

The findings in our study show that women who had delivered safely at home tended to plan to continue to deliver at home, and often doubted any advice to the contrary. These findings are supported by other studies from low-resource countries [20,34,38]. This could be reflected in the fact that previous experience of a safe, uncomplicated home delivery strengthens the individuals’ belief in the safety and norm of home deliveries. Positive experiences can also decrease perceptions of vulnerability to complications, as well as increase confidence in the TBAs representing the folk sector [28].

### The influence of interpersonal relationships

Decisions are rarely made by one individual, but are rather an amalgamation of the many people involved in the pregnant woman’s life. Each individual has, at least partly, different experiences and beliefs, which constitute a variety of explanatory models (30). These models, to varying degrees, will influence the woman in labour and act as barriers or facilitators in seeking certain forms of health care, findings similar to other studies from sub-Saharan Africa and Asia [34,35,41].

The multigravida women in this study appeared to have more autonomy and perceived control in relation to their deliveries. This could possibly be due to these women having their own experiences and perhaps carrying more “robust” explanatory models [28]. A more robust framework for understanding the different elements of the birth process may lead to a better ability to make and justify choices. In addition, a woman who has given birth before has already gone through the transitional period of being a young girl or woman to becoming a mother [42]. Most women in Sierra Leone belong to a secret society, where girls are initiated into womanhood making them eligible for marriage [43]. During this rite of passage from childhood to womanhood, women are circumcised and educated about pregnancy and childbirth [15,27]. The word secret is taken literally as women are not meant to discuss what takes place during the initiations with those who have not undergone the initiation themselves [15]. What is explained about the exact childbirth process remains unknown to outsiders.

In contrast to the multigravida women, most first-time mothers in this study lacked detailed knowledge of the childbirth process, which in turn affected their ability to make and justify choices during childbirth. The explanatory models [28] seemed to be based on hearsay or limited information from family members. The women were heavily reliant on the advice and support found within the popular sector, specifically their mothers or mother in laws, as well as the TBAs. A study by Bedford et al. [36] found a similar situation in rural Ethiopia where labour and childbirth were never discussed so as ‘not to frighten the expectant mother’ , causing total dependence on others. The health workers in the current study suggested that due to this situation first-time mothers are more likely to attend the hospital to deliver – a phenomenon also found in other studies from sub-Saharan Africa and Timor-Leste [34,38].

Our analysis confirmed findings from other studies regarding the particularly influential role TBAs can have within decision-making processes during childbirth [15-17,27]. TBAs are no longer officially integrated into the professional health sector nor recognised by the Government as skilled attendants, and are therefore considered part of the folk sector in this context [15,44,45]. Their experience, leadership role within the community and status within the secret societies implied that their opinions and advice are often well regarded and trusted. Furthermore, it is the role of the TBAs or female family members to inform the family about the progress of the labour, if complications occur and if additional care is required. The male members are particularly reliant on this information since they are not allowed near the birthing area. This influential role of the TBA’s has been documented in previous research conducted in Sierra Leone [15,17].

### Local explanatory models and responses to risk

Although pregnancy is seen as a normal condition in this rural setting, individuals and communities are aware of many different risks associated with both pregnancy and childbirth [27]. Beliefs about what causes a particular problem or risk will represent important elements of peoples explanatory models and strongly influence what type of treatment is sought, when, from where, or if additional assistance is sought at all.

In rural Sierra Leone it is the norm for several generations or a number of families to live together within one home [46]. Securing privacy, especially during childbirth, can therefore be difficult. Limiting the number of people assisting the woman ensured a certain level of confidentiality, a phenomenon also highlighted as important in Malawi [35]. Being exposed to a crowd was seen as a particularly difficult situation since women would be vulnerable to gossip as well as many people’s interpretations and advices. For example, an obstructed labour could be interpreted as a woman having behaved immorally during her pregnancy, which within the rural Sierra Leone context usually relates to infidelity [15]. In this situation advice from the crowd might be for the woman to confess to the infidelity in order to treat the obstruction, rather than seek assistance at the hospital.

Another perceived risk during childbirth was related to exposure to witchcraft. Fear of witchcraft complicating the delivery are in line with other studies from Sierra Leone and Malawi where women wait until labour is well established before attempting to access the hospital [15,17,35]. Explanatory models involving witchcraft may prevent a woman from seeking assistance; represent an incentive to seek care from a traditional provider; and act as an obstacle to, or delay her in seeking health services within the professional sector [47].

A systematic review by Moyer et al. [34] found that many women did not want to access treatment at the hospital during childbirth due to a fear of episiotomies. Participants in our study referred to a fear of automatically having an operation if delivering at the hospital. Women viewed having an operation as a potentially large risk to their health, thereby choosing not to access the hospital for treatment during labour. Distrust in health services actually being free, unreliable supplies of medication and equipment, and inadequacy of hospitals and clinics to cope with the increased number of women accessing them since the introduction of the FHCI were other concerns highlighted by participants. The perceived unreliability and reduced quality of care within the professional sector may imply that more reliable treatment is considered available within the folk or popular sectors. These findings are similar to a study conducted in Nigeria after free health care was initiated in 2008 [48].

Risks do not occur in isolation but are often considered and compared alongside other potential risks [49]. This study illustrates how women’s choice to deliver in the village is not a result of passive inaction or lack of knowledge about the potential risks she may face, but rather an active choice to reduce risks that she perceives as being of more importance. For example, a woman may consider the risk of witchcraft causing her labour not to progress being greater than the risk of bleeding, and will keep the onset of labour secret to reduce this more significant risk [15]. If however, the woman started to experience bleeding during the delivery, she may then decide to access care at the health facility, as this risk is now deemed a greater threat to her health. This syncretic use of the different health systems and health providers depending upon the identification and interpretation of different risk, illustrates a dynamic movement between different sectors as triggered by perceived needs [28].

Our study supported findings from previous studies conducted in Sierra Leone around the role of God as the ultimate decision maker [15,16]. This role was not static, often changing depending on circumstances, perceptions of risks, and strength of the individuals’ belief system. A number of participants referred to God as an explanation for specific problems during childbirth, especially when a plausible reason for a problem or condition was unknown. This lack of knowledge, combined with a reliance on God as an elucidation, strongly influences individuals’ explanatory models, justifying why particular actions are taken or treatment options chosen. Several participants expressed knowledge that the official advice from the Government was to deliver in the hospital. When the pregnant woman delivered in the village, the use of God as the ultimate decision-maker meant that the participants were not actively going against the official advice, instead their actions were explained by a higher power. God was also used to explain why some women die during childbirth. In this respect women were considered as ‘marked by God’ to die, thus their death could not be prevented, no matter what was done or where they were during childbirth. This fatalistic approach [49] to childbirth has also been documented in previous studies in Ethiopia and Sierra Leone [16,26].

### Explanatory models in flux

Most participants in this study described using both traditional medicines, in the form of native herbs or sorcery, and biomedical medicine, often described as ‘oporto’ medicine. Use of the different treatment options was flexible and interchangeable, with rarely a clear dichotomy between those who accessed health services from the professional sector and those who utilised the folk sector [28]. Instead medical syncretism [50] was demonstrated as participants dynamically moved between the different health sectors and health providers available to them. This type of flexible health seeking behaviour has been identified in previous studies conducted in Sierra Leone and Timor-Leste [15,16,38].

The medical pluralism that exists within the rural Sierra Leone context means that members of the community can choose between various options available depending upon the situation that they are currently in and how symptoms develop over time [50]. This can also depend upon how the different individuals interpret the “clinical realities”, and which explanations best fit their belief system around the cause of the condition or problem [28]. The indistinctness and frequent fluctuations in participants’ explanation for causes and appropriate treatment meant that treatment from all health sectors could be used concurrently. In addition, participants often viewed treatment from all sectors as having equal efficacy and value. Different treatments could be used to address different potential causes of the same problem, playing complementary rather than competitive roles [51]. Diverse and dynamic interpretation of risks by various individuals also implied that different health care systems could be accessed for the same problem. One example being convulsions during childbirth: if interpreted as caused by a demon, treatment would be sought from a traditional healer within the village. If the convulsions were associated with the woman being non-compliant in regards to advice during her antenatal appointments, treatment would be sought from within the professional sector. Most healthcare seekers negotiate various options available to them, thereby strategically obtaining the best possible treatment and management [28].

Perceived severity of a condition or symptoms was another feature that could influence and change the rationale of people’s health seeking behaviour. In this study the main symptoms motivating women to seek care specifically at the hospital were excessive bleeding or obstructed labour. Although these physical symptoms are often perceived as best treated by representatives from the biomedical sector, trust was also placed in the expertise found in the folk sector. This persistent trust in the folk sector probably relates to the fact that traditional medicines have been around for much longer, and at times have been the only form of health care available to many people, especially during the recent civil conflict [39]. However, many people have experienced that representatives of the biomedical professional sector are highly competent at handling acute, life-threatening situations that are often seen as impossible to be dealt with in the village. Therefore, care may immediately be sought from the professional sector in situations interpreted as life threatening, and where the cause is considered to be within the reach of biomedicine.

### Strengths and limitations

Findings from this study should be interpreted in light of several limitations. The literature review was limited to articles published in English and available via English-language search engines. This is of particular note since many of the West African countries are French speaking, and information and experiences coming from those neighbouring countries may be different to those described only in English. This language barrier was also apparent within the field with the first author being dependent upon an interpreter.

A mixed purposive sampling technique was used when selecting the participants, implying that they were not randomly selected nor were they necessarily representative of the whole community. In addition, those who were included may have been the more confident members of the community, or those with adequate time to participate. Although the project tried to capture a variety of perspectives from a number of different participants, the results may not be transferable to all rural areas of Sierra Leone nor to all low resource settings. However, if the contexts are similar regarding health beliefs, limited resources and infrastructure then the likeliness of the findings being transferable is larger.

Despite these limitations, there are a number of strengths worthy of note within this study. In order to understand women’s choices, one has to explore the processes she is going through, as well as map all the dynamic factors that interact and interrelate, and subsequently influence health seeking behaviour. The use of qualitative methods ensured that a deeper understanding of the decision-making process was gained. An explorative and flexible approach enabled new themes to be explored as they emerged. Due to the relatively long time in the field and the flexibility of the research team, fairly remote villages were included. This meant that views and experiences of communities in these secluded areas were included. Two different villages were purposively chosen to ensure variation with regards to geographical site and socio-demographic variables of the communities involved. This was deemed important so as to add more perspectives, which helps contrast and validate the findings. In addition to triangulation of research sites, this study also triangulated with respondent sources and methods so that a richer understanding about the issues and phenomena being studied could be obtained.

## Conclusions

The findings of this study demonstrate that the decision-making processes during childbirth in rural Sierra Leone are dynamic, intricate and need to be understood within the broader social context. Decisions are usually socially negotiated and rarely made independently. Past experiences, social expectations and relationships of those involved, as well as the risks perceived by the individual and their community are also influential. Individuals may have preferences for where to give birth and with whom assisting, but these are weighed up against the complexity of enabling, supportive and inhibitory factors that are present within the health care systems and social context.

Introduction of the Free Health Care Initiative has been a positive and significant approach towards improving access to healthcare for pregnant women. However, steps are needed to ensure that the most vulnerable and socially marginalised are included, especially the poor women living in isolated rural areas. The norm for many of these women is still to deliver at home, and removing them from their homes and responsibilities adds extra burdens for the women themselves. Since health facilities are often under-resourced and under-staffed, imposing sanctions such as the bylaws to ensure that women attend a health facility for a non-complicated delivery may not be the best strategy at present for communities in rural Sierra Leone. Bringing health services, including skilled birth attendants, closer to the communities may be a better option. Future initiatives need to be based on adequate knowledge of women’s preferences, cultural-specific traits, capabilities, perceptions of risk and the constraints in which they may live.

## Abbreviations

TBA: Traditional birth attendant
Oporto: Biomedical medicine
IDI: In-depth interview
FGD: Focus group discussion
FHCI: Free health care initiative
MMR: Maternal mortality ratio
